# Selective inhibition of HDAC6 promotes bladder cancer radiosensitization and mitigates the radiation-induced CXCL1 signalling

**DOI:** 10.1038/s41416-023-02195-0

**Published:** 2023-02-21

**Authors:** Yu-Chieh Tsai, Tzu-Yin Wang, Chia-Lang Hsu, Wei-Chou Lin, Jyun-Yu Chen, Jia-Hua Li, Yeong-Shiau Pu, Ann-Lii Cheng, Jason Chia-Hsien Cheng, Sheng-Fang Su

**Affiliations:** 1 Department of Oncology, National Taiwan University Hospital, Taipei, Taiwan; 2 Graduate Institute of Oncology, National Taiwan University College of Medicine, Taipei, Taiwan; 3 Department of Medical Research, National Taiwan University Hospital, Taipei, Taiwan; 4 Graduate Institute of Medical Genomics and Proteomics, National Taiwan University College of Medicine, Taipei, Taiwan; 5 Department of Pathology, National Taiwan University Hospital, Taipei, Taiwan; 6 Institute of Statistical Science, Academia Sinica, Taipei, Taiwan; 7 Department of Urology, National Taiwan University Hospital, Taipei, Taiwan; 8 Department of Medical Oncology, National Taiwan University Cancer Centre, Taipei, Taiwan; 9 YongLin Institute of Health, YongLin Scholar, National Taiwan University, Taipei, Taiwan

**Keywords:** Bladder cancer, Gene expression, Radiotherapy, Target identification, Cell invasion

## Abstract

**Background:**

Although trimodality therapy resecting tumours followed by chemoradiotherapy is emerged for muscle-invasive bladder cancer (MIBC), chemotherapy produces toxicities. Histone deacetylase inhibitors have been identified as an effective strategy to enhance cancer radiotherapy (RT).

**Methods:**

We examined the role of HDAC6 and specific inhibition of HDAC6 on BC radiosensitivity by performing transcriptomic analysis and mechanism study.

**Results:**

HDAC6 knockdown or HDAC6 inhibitor (HDAC6i) tubacin exerted a radiosensitizing effect, including decreased clonogenic survival, increased H3K9ac and *α*-tubulin acetylation, and accumulated γH2AX, which are similar to the effect of panobinostat, a pan-HDACi, on irradiated BC cells. Transcriptomics of shHDAC6-transduced T24 under irradiation showed that shHDAC6 counteracted RT-induced mRNA expression of CXCL1, SERPINE1, SDC1 and SDC2, which are linked to cell migration, angiogenesis and metastasis. Moreover, tubacin significantly suppressed RT-induced CXCL1 and radiation-enhanced invasion/migration, whereas panobinostat elevated RT-induced CXCL1 expression and invasion/migration abilities. This phenotype was significantly abrogated by anti-CXCL1 antibody, indicating the key regulator of CXCL1 contributing to BC malignancy. Immunohistochemical evaluation of tumours from urothelial carcinoma patients supported the correlation between high CXCL1 expression and reduced survival.

**Conclusion:**

Unlike pan-HDACi, the selective HDAC6i can enhance BC radiosensitization and effectively inhibit RT-induced oncogenic CXCL1-Snail-signalling, thus further advancing its therapeutic potential with RT.

## Introduction

Although radical cystectomy (RC) remains the gold standard for patients with muscle-invasive bladder cancer (MIBC) [1], bladder-preserving trimodality therapy (TMT), which combines maximal transurethral resection of bladder tumours followed by concurrent chemotherapy and radiotherapy (RT), has been shown to be an effective alternative to patients who are either not suitable surgical candidates or prefer organ preservation [2, 3]. A recent systematic review found similar overall survival but inferior cancer-specific survival in TMT patients compared with RC patients [4], and the chance of undergoing salvage cystectomy occurred in 10.7% of patients receiving TMT [5]. Nevertheless, the recommended chemotherapy regimens, such as cisplatin with fluorouracil or paclitaxel, fluorouracil with mitomycin C, or cisplatin alone (NCCN, preferred/2A) [6], have many well-recognised toxicities. Therefore, there is an unmet need to develop a more effective strategy to enhance the efficacy of RT in BC without the induction of unwanted toxicities.

We have previously reported that afatinib, an EGFR/HER-2 dual inhibitor, can radiosensitize BC cells by enhancing radiation-induced DNA damage [7]. Interestingly, histone acetylation has been regarded as a determinant of the radioresponse through mechanisms that regulate chromatin structure and gene expression [8]. By interfering with DNA damage signalling and repair pathways, inhibitors of histone deacetylases (HDAC) decrease the ability of tumour cells to repair radiation-induced DNA damage [9]. Several HDAC inhibitors (HDACi) have entered clinical trials for evaluation of efficacy and toxicity in combination with RT or chemoradiation [10, 11].

In BC, panobinostat, a hydroxamate pan-HDACi, has been demonstrated to have radiosensitizing activity through E3 ligase cellular inhibitor of apoptosis protein 2 (cIAP2)-mediated posttranscriptional downregulation of meiotic recombination 11 homologue (MRE11) [12, 13]. An in vivo study by Groselj et al. further demonstrated that panobinostat and RT inhibited bladder tumour growth in RT112 xenografts better than did RT alone without a significant increase in radiation toxicity in the normal tissue [14]. In addition, panobinostat showed clinical benefit in a phase I trial for advanced UC [15]. However, dose-limiting toxicities (DLTs) were reported in recurrent glioma patients who had received a high dose (30 mg) of panobinostat combined with stereotactic irradiation [16]. Therefore, the study of selective HDACis is encouraged, as specific HDACis may be more efficacious and have fewer systemic side effects [14, 17, 18].

HDAC6 is a structurally and functionally unique deacetylase that targets both histone and nonhistone substrates, such as heat shock protein (Hsp90), cortactin, peroxiredoxin, α-tubulin, and heat shock transcription factor-1 (HSF-1) [19, 20], resulting in diverse biological effects. HDAC6 has the ability to promote cell motility, migration and invasion [21, 22]. Selective inhibition of HDAC6 induces DNA damage, suppresses tumour proliferation and sensitises transformed cells to anti-cancer agents [23–25]. Moreover, HDAC6-specific inhibitors (HDAC6i) have entered clinical investigation as part of a combination regimen for anti-cancer activity [19]. Ricolinostat and citarinostat are selective HDAC6is that were found to enhance efficacy in relapsed multiple myeloma [26, 27]. Interestingly, an increasing number of studies, including preclinical models, have reported the immunoregulatory effect of HDAC6is on cancer suppression and prolonged survival with no significant toxicity [28, 29]. Nevertheless, there is limited investigation of the effects of HDAC6is on radioresponse, especially in treating BC.

In this study, we examined the role of HDAC6 and HDAC6 inhibition on BC radiosensitivity. We further performed transcriptomic analysis to elucidate the target genes affected by HDAC6 knockdown in T24 cells undergoing ionising radiation (IR). To better understand the clinical efficacy of selective HDAC6is combined with RT, we applied tubacin, a highly potent HDAC6i, to T24 cells and observed a repression of the RT-induced CXCL1 signalling pathway for cancer progression that was not present when applying panobinostat, suggesting a potential strategy of specifically targeting HDAC6 as a radiosensitizer for cancer treatment.

## Materials and methods

### Cell culture and irradiation

The human bladder carcinoma cell lines T24 and UMUC-3 were obtained from the American Type Culture Collection (ATCC, Manassas, VA), and BFTC909 cells were obtained from Bioresource Collection and Research Centre (BCRC, Taiwan). Cells were cultured in Roswell Park Memorial Institute (RPMI) 1640, Minimum Essential Medium (MEM) and Dulbecco’s modified Eagle’s medium (DMEM) supplemented with 10% foetal bovine serum (FBS) and penicillin/streptomycin. All cell lines were incubated at 37 °C in a humidified atmosphere containing 5% CO_2_. Cells were exposed to IR at doses ranging from 2.5 to 10 Gray (Gy) using a ^137^Cs γ-irradiator.

### Reagents

Panobinostat (LBH589) and tubacin were purchased from Selleck Chemicals (Houston, TX) and prepared at the indicated concentrations in dimethyl sulfoxide (DMSO). Monoclonal anti-CXCL1 and control IgG antibodies for neutralising treatment were acquired from R&D Systems (Minneapolis, MN).

### Cell viability assay

A total of 3 × 10^3^ cells were seeded onto 96-well plates and treated with panobinostat or tubacin at the indicated concentrations. Cell viability was assessed at 24 and 72 h by the Cell Counting Kit-8 (CCK-8) assay (Dojindo Molecular Technologies, Rockville, MD). The absorbance at 450 nm was measured with a microplate enzyme-linked immunosorbent assay reader (Epoch 2 Microplate Reader, BioTek Instruments, Winooski, VT).

### Clonogenic assay

Cells seeded on 6-well plates were irradiated at 0, 2.5, 5, 7.5 or 10 Gy with the indicated concentrations of panobinostat or tubacin. After 7 days of colony growth, cells were fixed with 20% methanol and stained with 0.25% crystal violet for 30 min. Colonies with >50 cells were counted under the whole microscope field.

### γH2AX immunofluorescence microscopy

A total of 1 × 10^5^ cells were plated on poly-D-lysine-coated chamber slides and exposed to 5 Gy irradiation in the presence or absence of 10 nM panobinostat. Following treatment for the indicated time intervals (0.5, 2, 4, 24 h), the cells were fixed in 4% paraformaldehyde in phosphate-buffered saline (PBS), permeabilized in 0.5% Triton X-100 in PBS and blocked in 5% bovine serum albumin (BSA). Then, the cells were incubated with primary rabbit anti-γH2AX antibody (Cell Signaling Technology, Danvers, MA) and mounted with mounting medium containing DAPI (Vector Laboratories, Burlingame, CA). γH2AX foci were examined under a fluorescence microscope with a 630X objective (Zeiss Axio Imager A1, Carl Zeiss, Germany) and counted using ImageJ.

### Western blot analysis

Proteins were extracted with RIPA lysis buffer containing protease and phosphatase inhibitors (Thermo Fisher Scientific, Waltham, MA), prepared in 5X sodium dodecyl sulfate (SDS) buffer, and analysed by SDS–PAGE (10% polyacrylamide gel). Following transfer to the polyvinylidene difluoride (PVDF) membrane, antibodies targeting HDAC1, HDAC2, HDAC3, HDAC4, HDAC5, HDAC6, acetyl-histone 3 (Lys9), acetyl-α-tubulin (Lys40), phospho-PTEN (Ser380/Thr382/383), PTEN, phospho-Akt (Ser473), Akt, Rad51, p21 Waf1/Cip1, Snail and GAPDH (Cell Signaling Technology, Danvers, MA) were used for immunoblotting (Supplementary Table S1). GAPDH was detected as the internal control. Protein bands were detected with an enhanced chemiluminescence (ECL) kit (Amersham International plc, Buckinghamshire, England) and analysed by MultiGel-21 (Topbio, Taiwan).

### Cell cycle analysis

A total of 5 × 10^5^ cells seeded on a 6-well plate were irradiated at 5 Gy in the presence of 10 nM panobinostat or 5 µM tubacin. At 16 h, the cells were fixed with 70% ethanol and labelled with propidium iodide (PI) using a cell cycle analysis kit (EZCell^TM^ Cell Cycle Analysis Kit, BioVision, Milpitas, CA) based on the manufacturer’s protocol. An LSRFortessa flow cytometer (Becton Dickinson, Franklin Lakes, NJ) was used. For each sample, 10000 events were examined, and the ratio of the distribution of cells within the cell cycle was determined by Modfit LT software.

### Transwell migration and invasion assays

Cells suspended in serum-free medium were seeded in the upper chamber of 24-well Transwell plates (8-μm pore, Corning Incorporated, Corning, NY) with or without 80 µg/mL Matrigel (Becton Dickinson, Franklin Lakes, NJ) coating. After 16 h and 20 h, migrated or invaded cells were fixed with methanol, stained with 0.25% crystal violet for 30 min, and counted under a microscope (Nikon Eclipse Ts2, Japan) using ImageJ.

### shRNA-mediated knockdown

A specific lentiviral vector carrying shRNA whose sequence targeted human HDAC6 (5′-CGGTAATGGAACTCAGCACAT-3′) and a control pLKO.1 vector were purchased from the RNA Technology Platform and Gene Manipulation Core (Academia Sinica, Taiwan). T24 cells at 80% confluence were subjected to lentivirus infection. After 72 h, the cells were selected with 2 mg/mL puromycin (Thermo Fisher Scientific, Waltham, MA).

### RNA-seq data processing

T24 cells subjected to HDAC6 knockdown following RT were prepared for RNA-seq. A Bioanalyzer 2100 was used for RNA quality control with an RNA 6000 LabChip kit (Agilent Technology, Santa Clara, CA). All RNA sample preparation procedures were carried out according to Illumina’s official protocol. Agilent’s SureSelect Strand-Specific RNA Library Preparation Kit was used for library construction followed by AMPure XP bead (Beckman Coulter, Brea, CA) size selection. The sequence was determined using Illumina sequencing-by-synthesis (SBS) technology (Illumina, San Diego, CA). Sequencing data (FASTQ reads) were generated using the pipeline established by Welgene Biotech, which is based on the base calling programme bcl2fastq v2.20 from Illumina. Read quality was evaluated by FastQC, and adaptor sequences were trimmed using cutadapt. Qualified reads were aligned to the human reference genome GRCh38 using STAR (v2.7.2) [30], and read counts for individual gene annotation based on GENCODE (v28) were subsequently determined using featureCounts [31]. Differential expression analysis was performed using NOISeq [32] with Trimmed Mean of *M*-values (TMM) normalisation. Genes with a differential expression probability > 0.4 were defined as DEGs. For pre-ranked GSEA, a ranking metric was calculated for each gene as *R* = rank * probability, where both rank and probability were determined by NOISeq. Pre-ranked GSEA was performed using a curated collection of gene sets from MSigDB. The significant gene sets were constructed as an enrichment map [33] using an in-house script and visualised by Cytoscape.

### Quantitative real-time PCR (qRT–PCR)

Total RNA was extracted using TRIzol (Thermo Fisher Scientific, Waltham, MA) and reverse transcribed using the HiSenScript™ RH(-) RT PreMix kit (iNtRON Biotechnology, Gyeonggi-do, Korea). Quantitative real-time PCR was conducted with primers (Supplementary Table S2, Integrated DNA Technologies, Coralville, IA) and SYBR Green (Thermo Fisher Scientific, Waltham, MA) using an Applied Biosystems StepOnePlus^TM^ real-time PCR system (Thermo Fisher Scientific, Waltham, MA). The thermal profile consisted of 1 cycle of 20 s at 95 °C followed by 40 cycles of 3 s at 95 °C and 30 s at 60 °C. GAPDH was used as the internal control.

### Enzyme-linked immunosorbent assay (ELISA)

A total of 1 × 10^5^ cells were plated onto a 6-well plate and subjected to 5 µM tubacin or 10 nM panobinostat treatment followed by 5 Gy irradiation. The conditioned medium was collected at 24 h and the CXCL1 protein level was determined using a Quantikine ELISA kit (Human CXCL1/GROα, R&D Systems, Minneapolis, MN) according to the manufacturer’s instructions.

### Immunohistochemistry staining

Tissue was fixed and embedded in paraffin blocks. Serial 4-µm paraffin sections were deparaffinized by EZ prep (Ventana Medical Systems, Tucson, AZ) and subjected to a 64-min pre-treatment using Cell Condition 1 solution (Ventana Medical Systems, Tucson, AZ). The slides were incubated with primary antibodies for 32 min using the automated Ventana Benchmark XT (Ventana Medical Systems, Tucson, AZ). Signals were detected with the Optiview DAB Detection Kit (Ventana Medical Systems, Tucson, AZ) according to the manufacturer’s protocol on an Olympus BX53 microscope (Olympus Corporation, Tokyo, Japan). All sections were counterstained with haematoxylin (Ventana Medical Systems, Tucson, AZ). Antibodies against GROα (Santa Cruz Biotechnology, Dallas, TX) are listed in Supplementary Table S1.

### Clinical specimen

Paraffin blocks from 40 patients with recurrent urothelial carcinoma who had received transurethral resection of bladder tumour (TURBT) dating from 2013 to 2017 were obtained from the Department of Pathology archives at National Taiwan University Hospital and examined by H&E staining for histological verification of disease status (Table 1). Most of them were classified as pathological T-stage pT3 (26/40). The study was approved by the Ethical Committee of National Taiwan University Hospital (IRB 201912011RINC).

**Table 1:** Clinicopathologic characteristics of 40 patients.

Variables	Number of cases	CXCL1 expression	High (*n* = 16)	*P*-value
		Low (*n* = 24)		
Age (years)				
≤65	21	14	7	
>65	19	10	9	0.3656
Gender				
Male	24	15	9	
Female	16	9	7	0.6926
Tumour position				
Bladder	16	10	6	
UTUC	24	14	10	0.7921
Pattern of progression				
Local recurrence	9	8	1	
Lymph node metastasis	22	10	12	
Visceral metastasis	9	6	3	0.0730
Lymph node metastasis				
Negative	18	14	4	
Positive	22	10	12	0.0379*

*IHC* immunohistochemistry, *UTUC* upper tract urothelial cancer.

*P  <  0.05.

### In vivo ectopic tumour model

Five- to 6-week-old male C57BL/6 mice were acquired from the National Laboratory Animal Centre and used for ectopic implantation. BC is more common in male than in female with a ratio of 3–4:1. Mice were randomly grouped, and ectopic tumours were established by subcutaneously injecting MB49 cells (2 × 10^6^) into the right hind limb of mice. Until the tumour size reached 100 mm^3^ (day 10), irradiation was applied in three 7.5-Gy fractions with a linear accelerator (Elekta Oncology System Ltd., Crawley, West Sussex, UK). Panobinostat and tubacin were prepared in sterile water containing DMSO, polyethylene glycol 300 (PEG 300) and polysorbate 80 (Tween 80) for intraperitoneal injection for 3 days, with daily doses of 10 and 25 mg/kg panobinostat and tubacin administered, respectively. Tumour volume was measured at 2-day intervals and calculated using the formula: tumour length (mm) x [tumour width (mm)]^2^ x 0.5. Mice were sacrificed when the tumour volume reached ~1500 mm^3^. All animal care, handling procedures, and experimental protocols were approved by the Committee of Experimental Animal Management at the College of Medicine, National Taiwan University.

### Statistical analysis

Data are presented as the mean with standard deviation (SD) for at least three independent experiments. Student’s *t*-test was used for paired comparisons, and differences were considered significant at a *P*-value < 0.05. The chi-square test was used to evaluate the relationship between two variables. The Kaplan–Meier method was used for survival analysis, and the log-rank test was used for comparison of survival data. Prism software version 9 (GraphPad software, San Diego, CA) was utilised for data plots.

## Results

### The pan-HDACi panobinostat enhances the radiosensitivity of bladder cancer cells

**Fig. 1:**
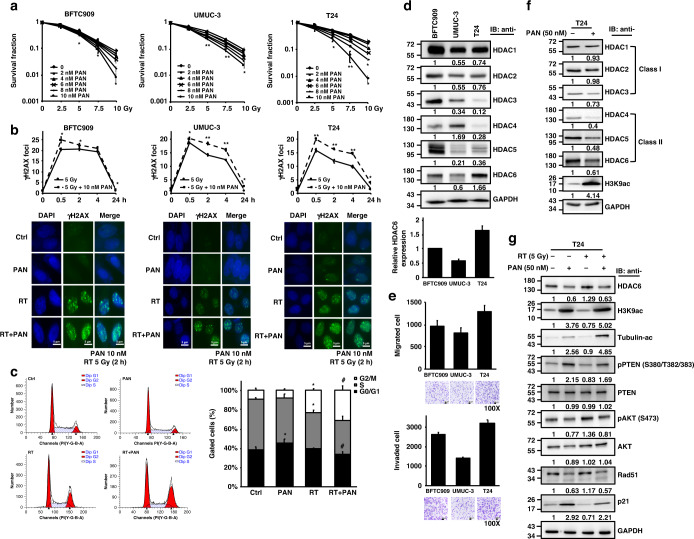
The radiosensitizing effect of the pan-HDAC inhibitor panobinostat in bladder cancer cell lines. **a** Clonogenic survival of human BC cells (BFTC909, UMUC-3, T24) treated with panobinostat (PAN) followed by irradiation (Gy). Colonies were stained with 0.25% crystal violet at day 7, and the survival rate was calculated. **b** Immunofluorescence staining with anti-γH2AX antibody was performed in cells with (PAN) or without (Ctrl) 10 nM panobinostat treatment followed by 5 Gy irradiation (RT). The average number of γH2AX foci per cell was analysed at 0, 0.5, 2, 4 and 24 h, and representative images at 2 h are shown below. **c** The cell cycle distribution of T24 cells treated with RT (5 Gy), panobinostat (10 nM), and a combination of RT and panobinostat (RT + PAN) was analysed at 16 h by flow cytometry. The percentages of cells within G0/G1, S, and G2/M phases are shown. Data are presented as the means ± SD from three independent biological replicates. Student’s *t*-test was used to determine significant differences. *P < 0.05; **P < 0.01, compared to control. #*P* < 0.05 compared to RT. **d** Western blotting analysis for class I (HDAC1, HDAC2, HDAC3) and class II (HDAC4, HDAC5/7, HDAC6) HDACs in BFTC909, UMUC-3 and T24 cells. GAPDH was used as the internal control. HDAC6 protein expression was quantified by ImageJ. **e** Cell migration and invasion abilities were examined under a microscope based on the numbers of stained migrated (16 h) and invaded (20 h) cells on the lower Transwell membrane. Representative images of BFTC909, UMUC-3 and T24 cells are shown below. **f** Western blotting for class I and class II HDACs and acetyl-histone H3 (Lys9) (H3K9ac) in T24 cells with or without 50 nM panobinostat treatment at 16 h. **g** Western blotting for HDAC6, H3K9ac, acetyl-α-tubulin, phospho-PTEN, PTEN, phospho-AKT, AKT, Rad51 and p21 expression in T24 cells treated with RT (5 Gy), panobinostat (50 nM), and the combination of RT and panobinostat at 16 h.

To study the dose effect of pan-HDACi on cell viability, we treated three human BC cell lines (T24, BFTC909 and UMUC-3) with different concentrations of panobinostat, which induced decreases in cell viability in all cell lines in a dose-dependent manner at 24 h and further decreases at 72 h (Supplementary Fig. S1). In combination with irradiation (2.5, 5, 7.5 and 10 Gy), clonogenic cell survival declined at day 7 under 2, 4, 6, 8 and 10 nM panobinostat treatment compared that after RT alone (Fig. 1a). In cells pre-treated with 10 nM panobinostat, a significant decrease in the survival fraction occurred starting at 5 Gy, indicating the radiosensitizing effect of panobinostat in BC cells. In addition, we utilised a γH2AX foci assay to estimate DNA damage in response to the combination treatment. Panobinostat (10 nM) promoted radiation-induced DNA damage between 0.5 and 24 h in all three cell lines (Fig. 1b). The fluorescence results showed an increased number of γH2AX foci in response to the combination of panobinostat and RT compared to RT alone. Additionally, radiation is known to induce G2/M cell cycle arrest, and panobinostat treatment further radiosensitized T24 cells to G2/M arrest (Fig. 1c).

To investigate how HDACs are targeted by panobinostat in BC, endogenous levels of HDAC proteins, including class I (HDAC1, HDAC2 and HDAC3) and class II (HDAC4, HDAC5 and HDAC6) HDACs, were first measured in BFTC909, UMUC-3 and T24 cells. The results showed that T24 cells had the highest baseline HDAC6 expression among the three cancer cell lines (Fig. 1d), in which HDAC6 protein expression was positively correlated with cell migration and invasion abilities (Fig. 1e), leading to the hypothesis that HDAC inhibition may reduce cancer cell progression ability. We found that panobinostat treatment (50 nM) decreased the expression of class II HDAC proteins, including HDAC6, and increased histone H3 lysine 9 acetylation (H3K9ac) in all cell lines (Fig. 1f; Supplementary Fig. S2). In RT/HDACi-treated T24 cells, although HDAC6, phosphorylated AKT and Rad51 protein expression increased after radiation, these effects were inhibited by combination treatment with panobinostat (Fig. 1g). Panobinostat treatment followed by RT also enhanced the phosphorylation of PTEN and the expression of p21, which are negative regulators of cell cycle progression (Fig. 1g).

### HDAC6 knockdown exhibits a radiosensitizing effect in bladder cancer cells

**Fig. 2:**
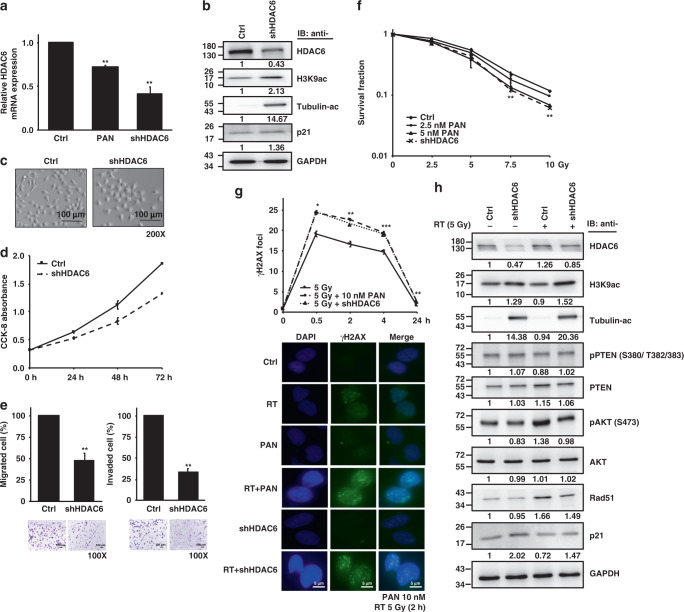
HDAC6 knockdown increases the radiosensitivity of T24 cells. **a** HDAC6 mRNA expression level in T24 cells with (shHDAC6) or without (Ctrl) HDAC6 knockdown as assessed by qPCR. Panobinostat (PAN) treatment was used for comparison. **b** Western blotting for HDAC6, H3K9ac, acetyl-α-tubulin and p21 expression in control and shHDAC6 T24 cells. GAPDH was used as the internal control. **c** Cell morphologies of the control and shHDAC6 T24 cells. Scale bar represents 100 μm. **d** Growth curves of the control and shHDAC6 T24 cells were determined by the CCK-8 assay at 24, 48 and 72 h. **e** Cell migration and invasion abilities were examined based on the numbers of stained migrated (16 h) and invaded (20 h) cells on the lower Transwell membrane as viewed under a microscope. Representative images of the control and shHDAC6 T24 cells are shown below. **f** Clonogenic survival of control and shHDAC6 T24 cells. Colonies were stained with 0.25% crystal violet at day 7, and the survival rate was calculated. **g** Immunofluorescence staining with an anti-γH2AX antibody was performed in control and shHDAC6 T24 cells subjected to RT (5 Gy). The average number of γH2AX foci per cell was analysed at 0.5, 2, 4 and 24 h, and representative images at 2 h are shown below. Data are presented as the means ± SD from three independent biological replicates. Student’s *t*-test was used for significant differences. *P < 0.05; **P < 0.01; ***P < 0.001. **h** Western blotting analysis in control and shHDAC6 T24 cells with or without RT (5 Gy) at 16 h.

Owing to the high expression of HDAC6 and high invasion/migration ability of T24 among the BC cells shown in Fig. 1d, e, we stably transfected shRNA against HDAC6 into T24 cells. HDAC6 mRNA (Fig. 2a) and protein (Fig. 2b) expression was measured by qPCR and Western blotting, respectively, to ensure the knockdown efficiency. Figure 2a shows a higher level of HDAC6 mRNA inhibition by HDAC6 knockdown than by panobinostat treatment, indicating specific inhibition of HDAC6 in shHDAC6 cells. T24 cells with HDAC6 knockdown had lower HDAC6 protein expression and higher H3K9ac levels than cells without HDAC6 knockdown as well as a marked increase in α-tubulin acetylation (Fig. 2b). Acetylation of tubulin regulates microtubule dynamics, cell motility and migration/invasion. The morphology of shHDAC6 T24 cells is shown in Fig. 2c. Compared to the control cells, shHDAC6 T24 cells seemed to become more adhesive, probably indicating loss of potential to migrate and invade. Moreover, knockdown of HDAC6 resulted in a decrease in cell growth (Fig. 2d) but not in cell viability (Fig. 2c). The shHDAC6 T24 cells showed a significant decrease in invasion/migration ability (Fig. 2e). A decreasing trend in invasion/migration was also observed in shHDAC6 BFTC909 and shHDAC6 UMUC-3 cells (Supplementary Fig. S3). Western blot analysis for EMT markers in BC cells with HDAC6 knockdown was shown in the Supplementary Fig. S4.

To examine the radiosensitizing effect of specific HDAC6 inhibition, phenotype studies were performed in T24 cells with HDAC6 knockdown. Under irradiation, decreased clonogenic survival was observed in shHDAC6 T24 cells at day 7 compared to that of control cells (Fig. 2f). In addition, HDAC6 knockdown in combination with RT induced the accumulation of γH2AX, which was more significant than that induced by RT alone (Fig. 2g), suggesting the enhanced efficacy of RT. Notably, HDAC6 knockdown abolished RT-induced protein expression of HDAC6, phosphorylated AKT and Rad51. Additionally, an increase in p21 and α-tubulin acetylation was shown in shHDAC6 T24 cells following RT compared to RT alone, although PTEN phosphorylation did not increase significantly (Fig. 2h). Altogether, the radiosensitizing effects caused by HDAC6 knockdown were similar to those of panobinostat shown in Fig. 1, suggesting that specific inhibition of HDAC6 functions similarly to a pan-HDACi in radiosensitizing BC cells.

### The selective HDAC6i tubacin radiosensitizes human bladder cancer cells

**Fig. 3:**
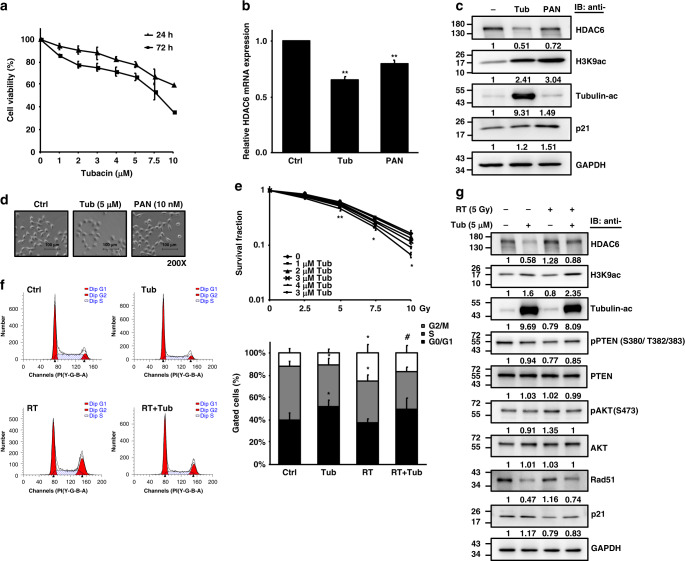
The radiosensitizing effect of the specific HDAC6 inhibitor tubacin in T24 cells. **a** The viability of T24 cells treated with tubacin (µM) was analysed at 24 and 72 h by the CCK-8 assay. **b** HDAC6 mRNA expression in T24 cells treated with 5 µM tubacin (Tub) was assessed by qPCR. Panobinostat (PAN) treatment was used for comparison. **c** Western blotting for HDAC6, H3K9ac, acetyl-α-tubulin and p21 expression in T24 cells treated with tubacin or panobinostat for 16 h. GAPDH was used as the internal control. **d** Cell morphologies of T24 cells treated with tubacin or panobinostat. Scale bar represents 100 µm. **e** Clonogenic survival of T24 cells treated with tubacin (Tub) followed by irradiation (Gy). Colonies were stained with 0.25% crystal violet at day 7, and the survival rate was calculated. **f** The cell cycle distribution of T24 cells treated with RT (5 Gy), tubacin (5 µM), and the combination of RT and tubacin (RT + Tub) was analysed at 16 h by flow cytometry. The percentages of cells within G0/G1, S and G2/M phases are shown. Data are presented as the means ± SD from three independent biological replicates. Student’s *t*-test was used for significant differences. *P < 0.05; **P < 0.01 compared to control. #*P* < 0.05 compared to RT. **g** Western blotting analysis for HDAC6, H3K9ac, acetyl-α-tubulin, phospho-PTEN, PTEN, phospho-AKT, AKT, Rad51 and p21 expression in T24 cells treated with RT (5 Gy), tubacin (5 µM), and the combination of RT and tubacin (RT + Tub) at 16 h.

To further investigate the radiosensitizing property of selective HDAC6is in clinical use, we treated T24 cells with tubacin at micromolar concentrations (Fig. 3a). This treatment resulted in a dose-dependent decrease in cell viability at 24 h and 72 h. The efficacy of tubacin in inhibiting the mRNA and protein expression of HDAC6 is shown in Fig. 3b, c, respectively. Compared to panobinostat treatment, tubacin treatment caused a higher degree of HDAC6 inhibition at the mRNA and protein levels and increased H3K9ac levels. Moreover, tubacin specifically induced α-tubulin acetylation (Fig. 3c). Unlike panobinostat, tubacin appeared to increase the adhesiveness of T24 cells after 24 h of treatment, similar to the morphology of shHDAC6 T24 cells (Fig. 3d).

In combination with irradiation, 5 µM tubacin significantly decreased clonogenic cell survival at day 7 compared to that after RT alone, suggesting that selective HDAC6i functions as a pan-HDACi radiosensitizer in BC cells (Fig. 3e). However, this effect did not seem to occur via induction of G2/M arrest in T24 cells (Fig. 3f). In addition, radiation-induced activation of HDAC6, AKT phosphorylation and Rad51 expression were repressed by tubacin while p21 protein expression and H3K9ac were elevated compared to those in response to RT alone. In concordance with HDAC6-knockdown T24 cells, tubacin followed by radiation did not affect the levels of PTEN phosphorylation (Fig. 3g). Tubacin also exerted a radiosensitizing effect on BFTC909 and UMUC-3 cells (Supplementary Fig. S5).

### Transcriptomic analysis of HDAC6 knockdown bladder cancer cells

**Fig. 4:**
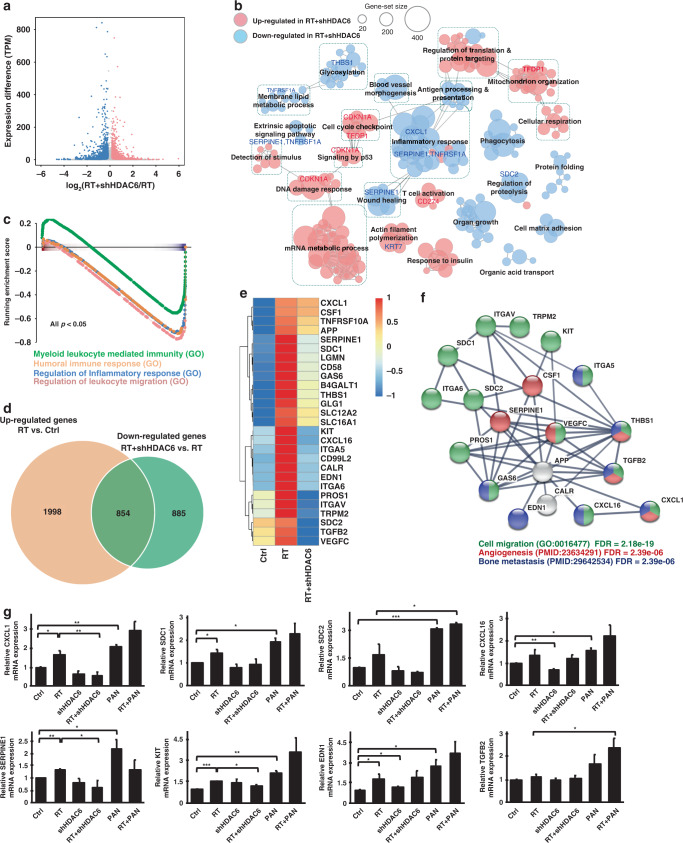
RNA-seq analysis of shHDAC6 T24 cells reveals that irradiation-induced genes were repressed by HDAC6 knockdown. **a** Scatter plot showing the log2 fold change for each gene between T24 cells with HDAC6 knockdown and subjected to radiation (RT + shHDAC6) and T24 cells subjected to radiation alone (RT) versus the differential expression in transcript per million (TPM). The red and blue dots denote the differentially expressed genes (DEGs) that are up- and downregulated, respectively, in RT + shHDAC6 cells compared to RT alone cells with a NOISeq probability >0.4. **b** Enrichment map of significantly enriched gene sets (FDR < 0.05) in RT + shHDAC6 T24 cells compared to RT alone cells. Nodes represent each gene set, and edges represent the connection of the similar gene sets in the network. Node size indicates the number of genes in the given gene set. **c** Gene set enrichment analysis (GSEA) of the DEGs in RT + shHDAC6 T24 cells shows enrichment in the inflammatory response. **d** The Venn diagram shows the overlap between the gene set of upregulated genes in T24 cells treated with RT compared to untreated cells (Ctrl) and the gene set of downregulated genes in RT + shHDAC6 T24 cells compared to RT alone cells. **e** Heatmap of gene expr**e**ssion from the overlapping gene set shown in **d** in the control, RT and RT + shHDAC6 T24 cells. **f** Protein–protein interaction (PPI) analysis of the gene set shown in **e**. **g** mRNA levels of CXCL1, SERPINE1, SDC1, KIT, EDN1, SDC2 CXCL16 and TGFB2 were validated by qPCR in control and shHDAC6 T24 cells treated with or without 5 Gy irradiation. 10 nM panobinostat was used for comparison. Data are presented as the means ± SD from three independent biological replicates. Student’s *t*-test was used for significant differences. *P < 0.05; **P < 0.01; ***P < 0.001.

We further performed RNA-seq analysis to determine the potential target genes under the regulation of HDAC6 in T24 cells exposed to IR. The data showed 12342 upregulated and 8705 downregulated differentially expressed genes (DEGs) in shHDAC6 T24 cells treated with RT (RT + shHDAC6) compared to parental T24 cells treated with RT (Fig. 4a). Upregulated DEGs were significantly enriched in DNA damage response, cell cycle checkpoint, p53 signalling, mitochondrion organisation and T cell activation, while downregulated DEGs were enriched in inflammatory response, wound healing, membrane lipid metabolic process, regulation of proteolysis and apoptotic signalling (Fig. 4b). We validated the mRNA expression of genes involved in these pathways using real-time qPCR and found upregulation of CDKN1A and downregulation of CXCL1, SERPINE1, SDC2 and TNFRSF1A in RT + shHDAC6 T24 cells compared to RT alone cells (Supplementary Fig. S6). Gene set enrichment analysis (GSEA) also demonstrated the key pathways regulated by HDAC6 knockdown under irradiation (Supplementary Fig. S7); among them, we noted that genes that were highly downregulated in RT + shHDAC6 T24 cells were significantly enriched in inflammatory and immune responses (Fig. 4c, *P* < 0.05).

Furthermore, to examine the impact of HDAC6 inhibition on the efficacy of RT, we analysed 2852 DEGs that were upregulated in RT-exposed T24 cells (RT) in comparison with untreated cells (Ctrl). We also found 1739 DEGs downregulated in the RT + shHDAC6 sample compared to RT-exposed T24 cells. By overlapping the two sets of genes, 854 radiation-induced DEGs were repressed when HDAC6 was knocked down (Fig. 4d). On the other hand, DEGs that were downregulated by radiation and induced by HDAC6 knockdown are shown in Supplementary Fig. S8. Moreover, among the 854 DEGs, those related to migration were identified and highlighted in the heatmap (Fig. 4e). The protein–protein interaction (PPI) analysis showed that the genes that were induced by irradiation and repressed by HDAC6 knockdown were significantly enriched in cell migration, angiogenesis and bone metastasis (Fig. 4f). This indicates that knockdown of HDAC6 may repress the expression of RT-induced genes that are associated with invasion and migration, including CXCL1, SERPINE1, SDC1, KIT, SDC2, EDN1, CXCL16, TGFB2, VEGFC, ITGA6 and CD58. The real-time qPCR data showed that RT did increase the mRNA expression of these genes involved in tumour progression (Fig. 4g; Supplementary Fig. S9); however, the increases were counteracted in RT + shHDAC6 T24 cells. We also investigated the impact of panobinostat on these target genes. Surprisingly, we found that in contrast to the effects of HDAC6i, the pan-HDACi panobinostat increased the expression of genes that had been induced by RT, indicating that HDAC6 inhibition may have a higher specificity and better efficacy against BC. An additional RNA-seq analysis (Supplementary Fig. S10) of cells treated with tubacin in combination with RT showed that the effect of tubacin treatment tends to be similar to the effect of shHDAC6, supporting the results obtained using shHDAC6 (Supplementary Fig. S11).

### The selective HDAC6i inhibits the expression of CXCL1, which is induced in irradiated bladder cancer

**Fig. 5:**
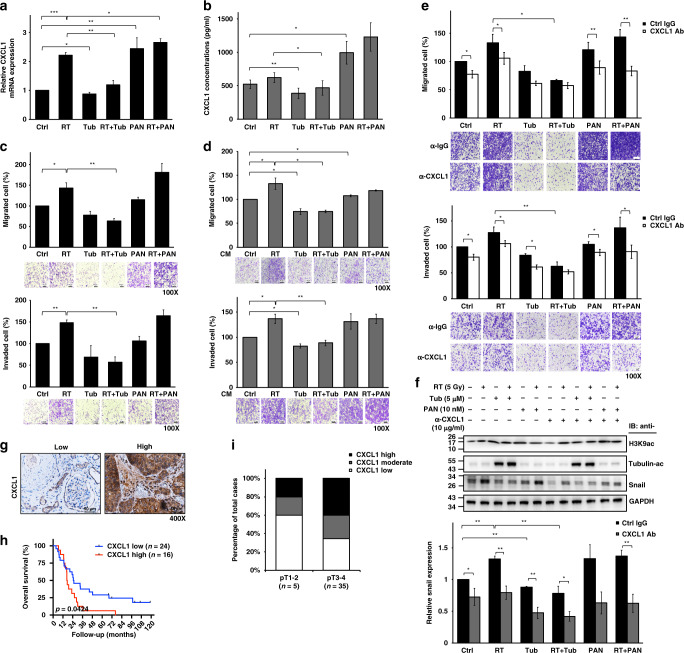
Tubacin represses RT-induced oncogenic CXCL1 signalling. **a** CXCL1 mRNA expression in T24 cells with or without 5 µM tubacin (Tub) treatment followed by 5 Gy irradiation (RT) was assessed by qPCR. Panobinostat (PAN, 10 nM) was used for comparison. **b** CXCL1 protein levels in conditioned medium from T24 cells with or without 5 µM Tub treatment followed by 5 Gy irradiation were measured by ELISA at 24 h. **c** Migration and invasion assays of T24 cells with or without 5 µM Tub treatment followed by 5 Gy irradiation. Migrated and invaded cells on the lower Transwell membrane were stained and calculated at 16 and 20 h, respectively. Representative images are shown below individually. **d** Migration and invasion assays of T24 cells treated with conditioned medium (CM) from cells with or without 5 µM Tub treatment followed by 5 Gy irradiation. Migrated and invaded cells on the lower Transwell membrane were stained and calculated at 16 and 20 h, respectively. Representative images are shown below individually. **e** Migration and invasion assays of Tub- and RT + Tub-treated T24 cells in the presence of 10 µg/mL anti-CXCL1 or control IgG treatment. Panobinostat (PAN)- and RT + PAN-treated cells were used for comparison. Migrated and invaded cells on the lower Transwell membrane were stained and calculated at 16 and 20 h, respectively. Representative images are shown below individually. **f** Western blotting for H3K9ac, acetyl-α-tubulin and Snail in Tub- and RT + Tub-treated T24 cells with or without anti-CXCL1 treatment at 24 h. Panobinostat (PAN)- and RT + PAN-treated cells were used for comparison. Snail protein expression was quantified by ImageJ. Data are presented as the means ± SD from three independent biological replicates. Student’s *t*-test was used for significant differences. *P < 0.05; **P < 0.01; ***P < 0.001. **g** Immunohistochemical staining for CXCL1 in tumour tissues obtained from urothelial carcinoma patients. Representative images are shown, and brown precipitates indicate positive signals. Scale bar represents 50 µm. **h** Overall survival analysis of 40 urothelial carcinoma patients categorised by CXCL1 protein levels. Statistical differences were assessed by the log-rank (Mantel–Cox) test. **i** Categorisation of CXCL1 expression in patients with high (pT3-4) and low (pT1-2) T stages.

CXCL1 plays a critical role in invasive BC and represents a promising biomarker for patient outcome prediction [34–36]. As a relatively high expression of CXCL1 was detected in T24 cells compared to BFTC909 and UMUC-3 cells (Supplementary Fig. S12), we applied tubacin to RT-exposed T24 cells to investigate the impact of a selective HDAC6i on CXCL1 expression. We found that cellular CXCL1 mRNA (Fig. 5a) and secreted CXCL1 protein (Fig. 5b) expression was increased by RT, but these increases were significantly abrogated when cells were treated with tubacin. In contrast, panobinostat further increased CXCL1 expression in T24 cells following IR exposure. To explore whether such CXCL1 induction results in tumorigenic phenotypes, we evaluated the migration and invasion abilities of RT-exposed T24 cells treated with tubacin or panobinostat. Cell viability was not affected by treatment with tubacin, panobinostat or the combination with RT (Supplementary Fig. S13). Notably, radiation-enhanced migration and invasion of T24 cells were reduced significantly in combination with tubacin, even to a lower extent than those of untreated T24 cells. However, this reductive effect did not occur when combined with panobinostat (Fig. 5c). Panobinostat further increased the radiation-enhanced migration and invasion of T24 cells. This phenomenon also occurred in BFTC909 and UMUC-3 cells (Supplementary Fig. S14). Although the pan-HDACi panobinostat sensitised BC cells to radiation and increased DNA damage, as shown in Fig. 1, it may cause higher toxicity and did not suppress radiation-induced CXCL1 expression, leading to increased migration and invasion. In addition, we collected conditioned medium (CM) from treated and untreated cells and performed cell migration and invasion assays on naïve T24 cells. The results showed that radiation-stimulated migration and invasion abilities were abolished significantly by CM from irradiated cells treated with tubacin but not by CM from irradiated cells treated with panobinostat (Fig. 5d).

To further clarify whether RT-induced CXCL1 expression is the key regulator contributing to BC malignancy, a monoclonal antibody against CXCL1 was used in T24 cells. Migration and invasion abilities, which were enhanced by cotreatment with panobinostat and radiation, were significantly suppressed by CXCL1 antibody neutralisation, but nearly no effect was observed in RT-exposed T24 cells treated with tubacin (Fig. 5e). We also studied the expression of Snail [37], a direct target of CXCL1, and showed that the high protein level of Snail under combined panobinostat and RT was markedly repressed by CXCL1 inhibition (Fig. 5f). In cells treated with tubacin and radiation, blocking CXCL1 further decreased Snail protein expression. The results showed the consistency of CXCL1 function with the results of migration and invasion assays shown in Fig. 5e.

### CXCL1 expression in urothelial tumour samples is associated with patient survival

Next, we investigated the clinical relevance of CXCL1 by collecting tumour tissues from 40 recurrent urothelial carcinoma patients who received TURBT but did not undergo radiation (Table 1). Immunohistochemical staining for CXCL1 was performed to stratify patients with high or low CXCL1 expression (Fig. 5g). The Kaplan–Meier analysis revealed that patients with higher levels of CXCL1 experience shorter overall survival (Fig. 5h, *P* = 0.04). CXCL1^high^ was also correlated with a high pT stage (Fig. 5i) and lymph node metastasis (Table 1), supporting the malignant role of CXCL1 and the therapeutic potential of CXCL1 inhibition. Use of a selective HDAC6i not only enhanced the radiosensitivity of T24 cells but also suppressed RT-induced CXCL1 expression. However, a larger cohort of patients undergoing RT treatment may be required in future studies if possible.

### The HDAC6i tubacin exerts a radiosensitizing effect on tumour growth in vivo

We established an in vivo ectopic tumour model to examine the radiosensitizing effect of the selective HDAC inhibitor tubacin. MB49 cells (2 × 10^6^) were injected subcutaneously into 5- to 6-week-old male C57BL/6 mice, and mice were monitored until the tumour size reached 100 mm^3^. Radiation was administered in 3 continuous 7.5-Gy fractions, and panobinostat or tubacin was administered simultaneously via intraperitoneal injection for 3 days. The tumour volume and mouse body weight were measured at 2-day intervals (Supplementary Fig. S15a). The tumour size was significantly reduced by cotreatment with radiation and tubacin (RT + Tub) on day 19 compared to mice treated with RT alone (Supplementary Fig. S15b). In the experimental period, the body weight of each mouse group was not significantly different (Supplementary Fig. S15c).

## Discussion

Conventionally, chemotherapy used in TMT functions as a radiosensitizing agent as well as systemic treatment for any micrometastatic disease [38]. Accumulated studies have shown an improved RT response while minimising overt toxicities in BC by combining tyrosine kinase inhibitors (TKIs), targeted hypoxia or angiogenesis molecules, epigenetic modifiers, and other drugs [39]. Our study is the first to demonstrate the radiosensitizing effect of a specific HDAC6i on BC. Our results also revealed an important issue: selective HDAC6is could suppress tumour migration and invasion by inhibiting CXCL1 induction in irradiated cancer.

Although the pan-HDACi panobinostat has been approved in patients with pre-treated multiple myeloma, it significantly increased the risk of thrombocytopenia, lymphopenia and diarrhoea in the pivotal PANORAMA1 phase III study [40]. These toxicities were exacerbated when panobinostat is combined with RT, especially for pelvic organs such as the bladder. In a phase I study combining panobinostat with RT for patients with inoperable stage III non-small-cell lung cancer, the incidence of grade 3/4 lymphopenia was 67% and that of grade 3/4 thrombocytopenia was 33% [41]. On the other hand, the selective HDAC6i ricolinostat revealed a favourable safety profile in a phase I/II study of myeloma despite no currently available randomised trial for comparison [27].

Here, we showed that specific inhibition of HDAC6 increased BC radiosensitivity and the accumulation of γH2AX, similar to the effect of panobinostat. However, the selective HDAC6i tubacin did not induce G2/M phase arrest in BC cells like panobinostat [12]. This result is consistent with a previous report that tubacin has less of an effect on DNA synthesis or the cell cycle [42], indicating the potential lower toxicity of HDAC6i for clinical use. Importantly, tubacin inhibited the RT-induced migration and invasion of BC cells, which differs from panobinostat. Although pan-HDAC inhibitors have been approved for use in the clinic, they may have higher toxicity, as panobinostat may elicit adverse effects, including upregulating RT-induced CXCL1 expression. Panobinostat has been reported to be capable of suppressing the migration, invasion and metastasis of thyroid [43] and liver [44] cancers, but a recent study revealed that 10 nM panobinostat could induce invasion in prostate cancer cells [45]. Our data showed that 10 nM panobinostat had little effect on BC migration and invasion, but the effect rose significantly when combined with RT. This phenomenon may relate to a pro-survival response to sublethal RT [46].

HDAC6 is a microtubule-associated deacetylase and has a crucial role in cell motility and migration [47]. A previous study showed that HDAC6 could promote BC cell migration and invasion [21]. Specific inhibition of HDAC6 results in acetylation of HSP90 [48], which regulates malignant transformation. In addition, from our RNA sequencing data, we identified a gene set that was upregulated by RT yet suppressed by HDAC6 inhibition. These genes, including CXCL1, SDC1, SDC2, EDN1, and KIT, are involved in invasion, migration, angiogenesis, inflammation, metastasis and tumour progression and have been identified as therapeutic targets in several types of cancer: CXCL1 [49, 50], SERPINE1 (PAI-1) [51, 52], SDC1 [53, 54], KIT [55], EDN1 [56], SDC2 [57], TGFB2 [58, 59], ITGA6 [60], VEGFC [61], and CD58 [62]. Among them, CXCL1 (C–X–C motif ligand 1), also known as growth-regulated oncogene α, is a member of the CXCL class of chemokines with angiogenic properties [63]. CXCL1 produced by tumour-associated macrophages in malignancies could form a pre-metastatic niche to promote metastasis [64]. In BC, CXCL1 has been recognised as a marker for tumour invasion [34]. Moreover, CXCL1 signalling in the tumour microenvironment is associated with tumour progression, tumour recurrence and drug resistance; [65] therefore, targeting CXCL1 signalling is a potential therapeutic approach for human BC. The HDAC6i used in our study inhibited CXCL1.

In conclusion, we discovered that HDAC6is function as more than radiosensitizers in BC. Selectively targeting HDAC6 also blocks CXCL1 signalling to advance anti-cancer therapy by repressing tumour invasion and migration.

## Supplementary information

Supplementary Information

## Data Availability

The RNA-seq data generated in this study are publicly available in Gene Expression Omnibus (GEO) at GSE197659.
