# Supplementary material for: Selective inhibition of HDAC6 promotes bladder cancer radiosensitization and mitigates the radiation-induced CXCL1 signalling
Source: Br J Cancer. 2023 Feb 21;128(9):1753–64. doi: 10.1038/s41416-023-02195-0 (PMC10133394; doi:10.1038/s41416-023-02195-0)
Supplement: Supplementary file 1 — Supplementary Information [file 41416_2023_2195_MOESM1_ESM.pdf]

Supplementary Information

Selective inhibition of HDAC6 promotes bladder cancer radiosensitization and mitigates the radiation-induced CXCL1 signalling

Yu-Chieh Tsai, Tzu-Yin Wang, Chia-Lang Hsu, Wei-Chou Lin, Jyun-Yu Chen, Jia-Hua Li, Yeong-Shiau Pu, Ann-Lii Cheng, Jason Chia-Hsien Cheng, Sheng-Fang Su

Contents:

Supplemental Figures 1 to 15

Supplemental Figure Legends 1 to 15

Supplemental Tables 1 to 2

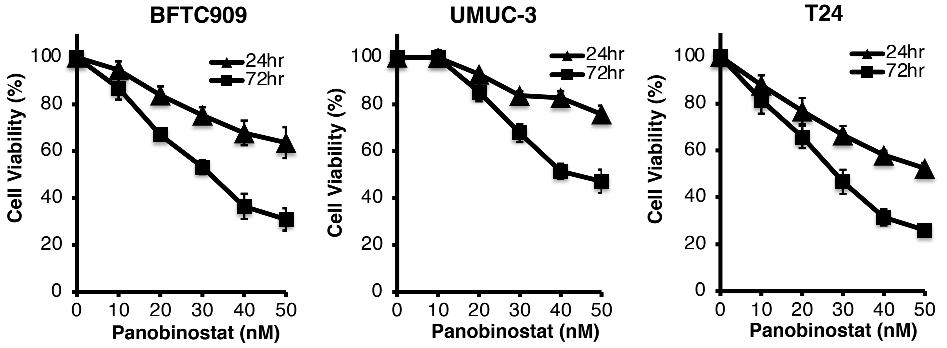

Figure S1. The viability of BFTC909, UMUC-3 and T24 cells treated with panobinostat (nM). The half maximal inhibitory concentration (IC_{50}) of panobinostat in different bladder cancer cell lines were analyzed at 24 and 72 hours by the CCK-8 assay. Data are presented as the means ± SD from three independent biological replicates.

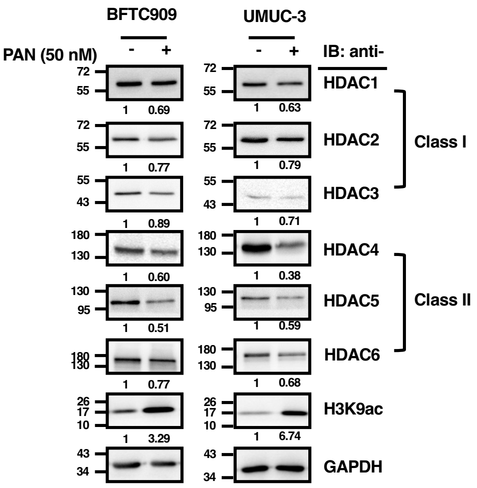

Figure S2. Protein expression of HDACs treated with panobinostat in bladder cancer cell lines. Western blotting analysis for class I (HDAC1, HDAC2, HDAC3) and class II (HDAC4, HDAC5, HDAC6) HDACs, and acetyl-histone H3 (Lys9) (H3K9ac) in BFTC909 and UMUC-3 cells with or without 50 nM panobinostat (PAN) treatment for 16 hours. GAPDH was used as the internal control.

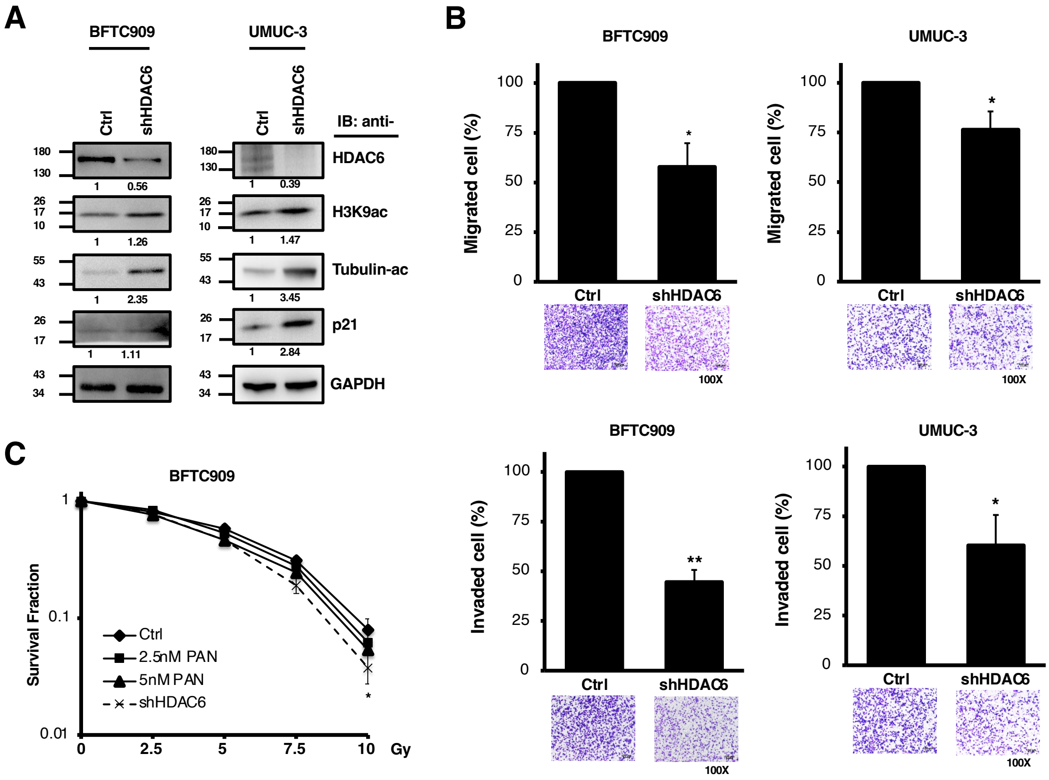

Figure S3. **HDAC6 knockdown resulted in decreased migration and invasion abilities of BFTC909 and UMUC-3 cells. a** Western blots showing the levels of HDAC6, H3K9ac, acetyl-α-tubulin and p21 in control and shHDAC6 BFTC909 cells. GAPDH was used as the internal control. **b** Cell migration and invasion abilities were examined based on the numbers of stained migrated (16 hours) and invaded (20 hours) cells on the lower surface of the Transwell membrane viewed under a microscope. Representative images of the control and shHDAC6-transfected cells (left panel: BFTC909 cells; right panel: UMUC-3 cells) are shown below. **c** Clonogenic survival of control and shHDAC6 BFTC909 cells. Colonies were stained with 0.25% crystal violet at day 7, and the survival rate was calculated. Data are presented as the means ± SD from three independent biological replicates. Student's t test was used to determine significant differences. * $P < 0.05$; ** $P < 0.01$.

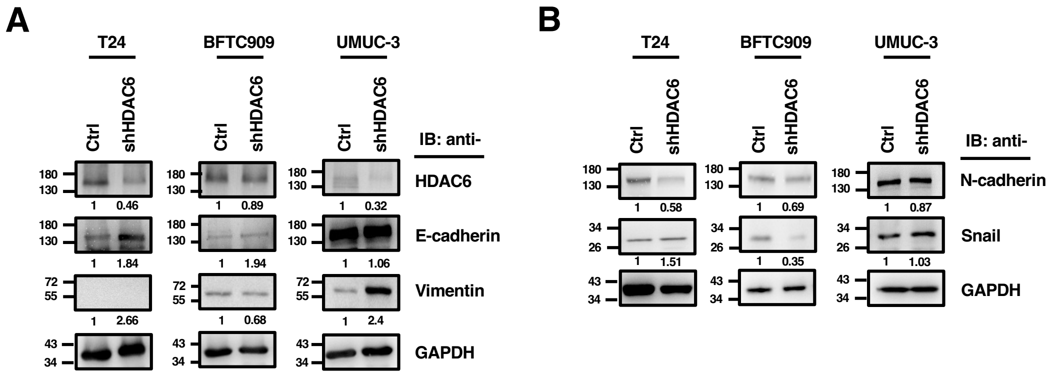

Figure S4. Protein expression of EMT markers in HDAC6 knockdown bladder cancer cell lines. Western blot analysis of E-cadherin, Vimentin, N-cadherin and Snail levels in T24, BFTC909 and UMUC-3 cells with (shHDAC6) or without (Ctrl) HDAC6 knockdown. GAPDH was used as the internal control.

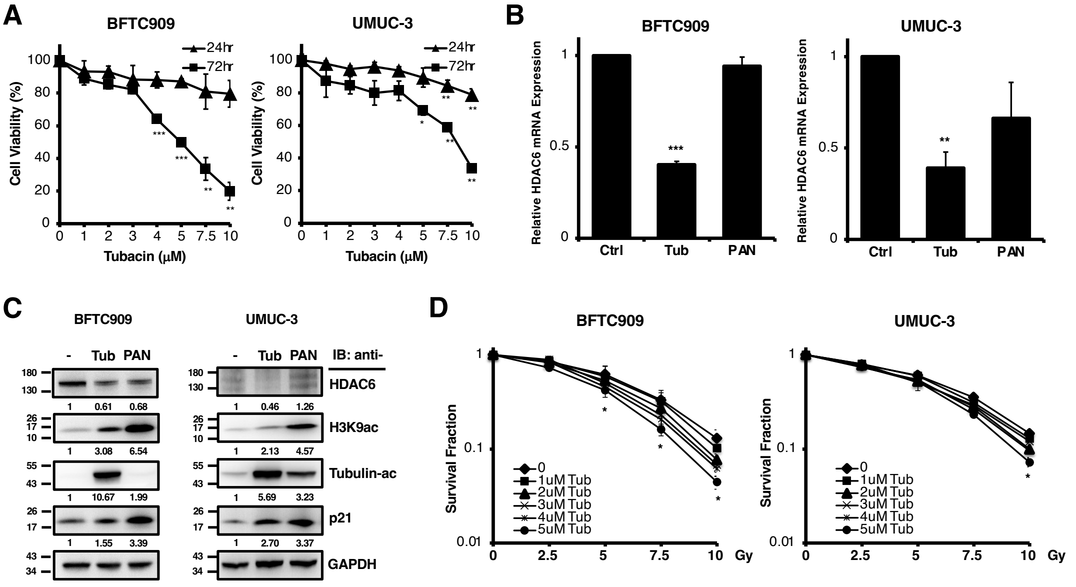

Figure S5. The radiosensitizing effect of the HDAC6-specific inhibitor tubacin on BFTC909 and UMUC-3 cells. **a** The viability of BFTC909 and UMUC-3 cells treated with different concentrations of tubacin (μM) was analyzed at 24 and 72 hours using the CCK-8 assay. **b** HDAC6 mRNA expression in BFTC909 and UMUC-3 cells treated with tubacin (Tub) was assessed by qPCR. Panobinostat (PAN) treatment was used for comparison. **c** Western blots showing the levels of HDAC6, H3K9ac, acetyl-α-tubulin and p21 in BFTC909 and UMUC-3 cells treated with tubacin (Tub) or panobinostat (PAN) for 16 hours. GAPDH was used as the internal control. **d** Clonogenic survival of BFTC909 and UMUC-3 cells treated with tubacin (Tub) followed by irradiation (Gy). Colonies were stained with 0.25% crystal violet on day 7, and the survival rate was calculated. Data are presented as the means ± SD from three independent biological replicates. Student's t test was used to determine significant differences. * $P < 0.05$, ** $P < 0.01$; *** $P < 0.001$, compared to the control.

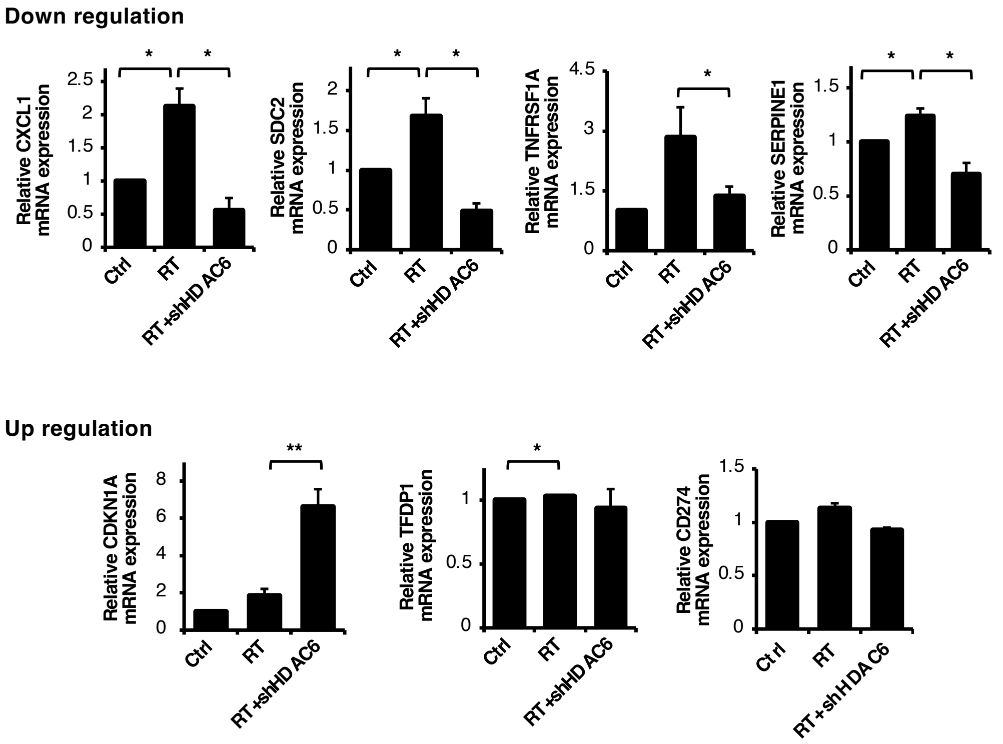

Figure S6. Validation of differentially expressed genes (DEGs) up- and downregulated, respectively, in RT+shHDAC6 cells compared to RT alone cells. mRNA levels of DEGs were analyzed by qPCR in control (Ctrl) T24 cells treated with or without 5 Gy irradiation (RT), and shHDAC6 T24 cells treated with RT (RT+shHDAC6). Data are presented as the means ± SD from three independent biological replicates. Student's *t* test was used for significant differences. * $P < 0.05$; ** $P < 0.01$.

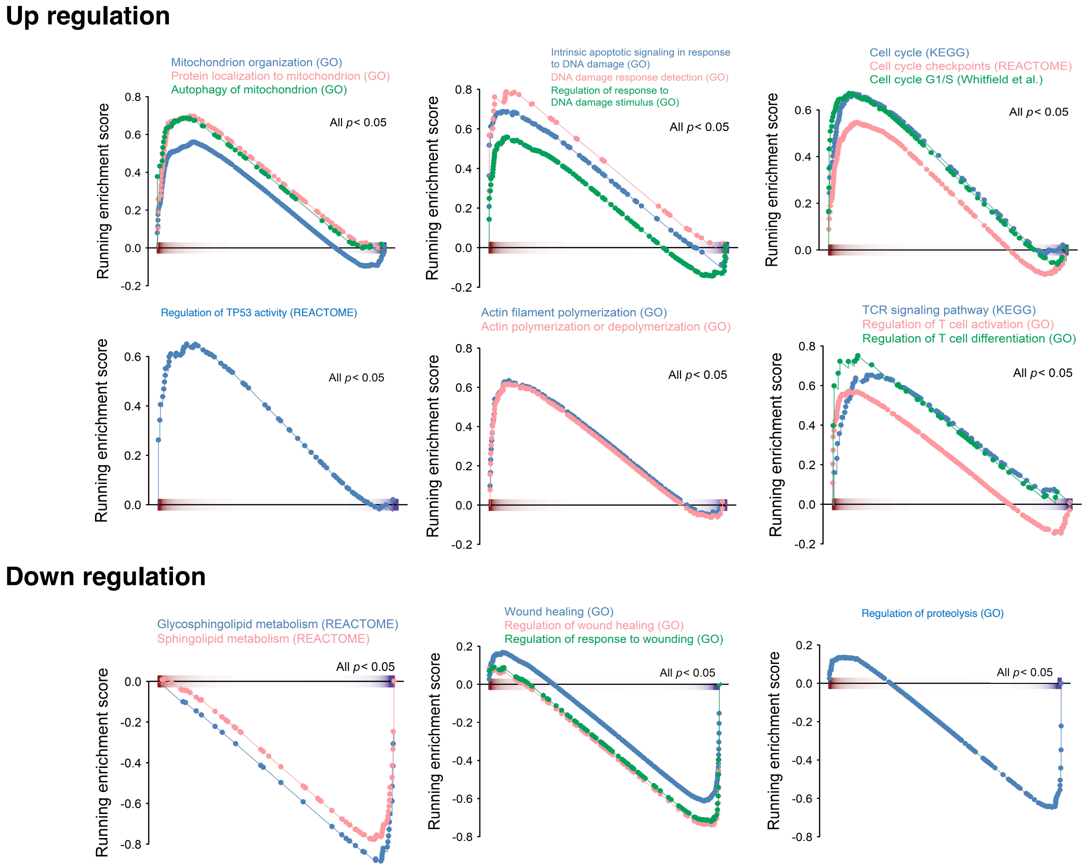

Figure S7. Gene set enrichment analysis (GSEA) of the DEGs in irradiated T24 with HDAC6 knockdown. GSEA enrichment plots for pathways of mitochondrion organization, DNA damage response, cell cycle checkpoint, signalling by TP53, actin filament polymerization, T cell activation; and membrane lipid metabolism, wound healing and proteolysis in RT+shHDAC6 T24 cells compared to RT alone cells.

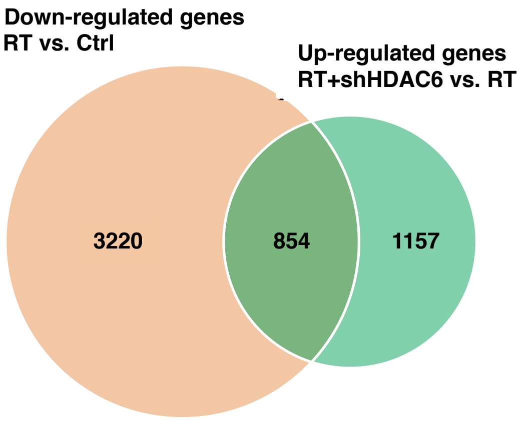

Figure S8. Numbers of irradiation-downregulated genes that were increased by HDAC6 knockdown in T24 cells. The Venn diagram shows the overlap between the gene set of downregulated genes in T24 cells treated with RT compared to untreated cells (Ctrl) and the gene set of upregulated genes in RT+shHDAC6 T24 cells compared to RT alone cells.

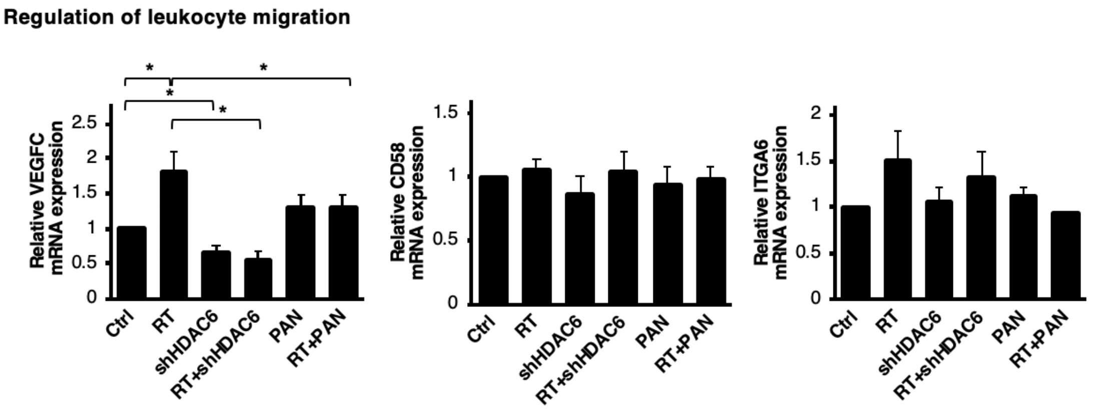

Figure S9. Validation of differentially expressed genes (DEGs) in RT+shHDAC6 cells compared to RT alone cells. mRNA levels of VEGFC, CD58 and ITGA6 were analyzed by qPCR in control and shHDAC6 T24 cells treated with or without 5 Gy irradiation (RT). 10 nM panobinostat (PAN) was used for comparison. Data are presented as the means ± SD from three independent biological replicates. Student's t test was used for significant differences. * $P < 0.05$.

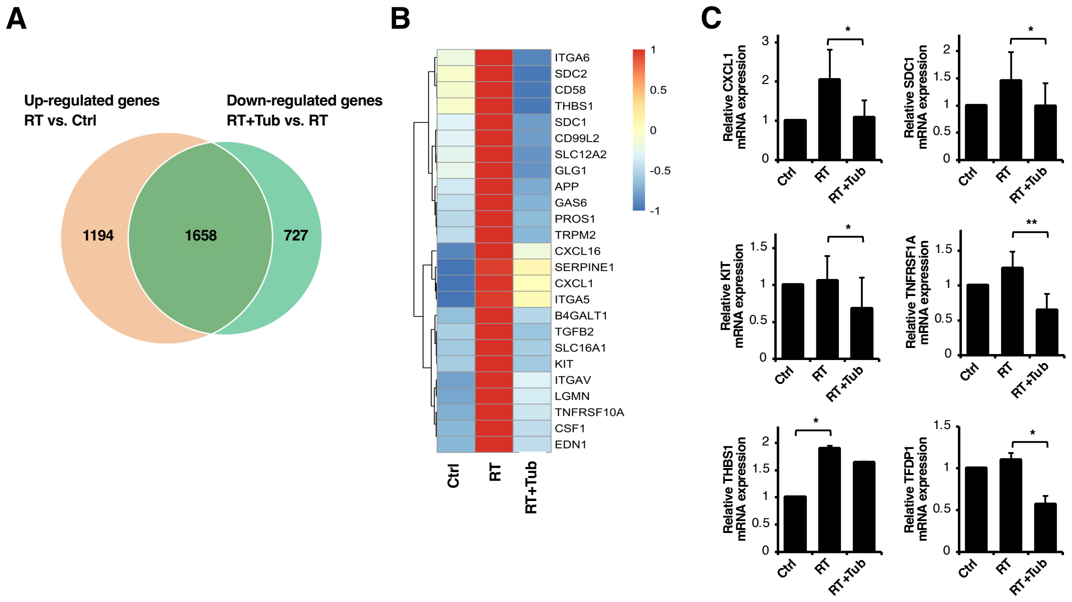

Figure S10. RNA-seq analysis of T24 cells treated with tubacin, an HDAC6 inhibitor. a The Venn diagram shows the overlap between the gene set of upregulated genes in T24 cells treated with RT compared to untreated cells (Ctrl) and the gene set of downregulated genes in T24 cells treated with radiation and tubacin (RT+Tub) compared to that treated with RT alone. **b** Heatmap of gene expression from the overlapping gene set shown in **a** in the control, RT and RT+Tub T24 cells. **c** q-PCR validation of DEG genes in control (Ctrl) T24 cells treated with or without 5 Gy irradiation (RT), and T24 cells treated with tubacin and RT (RT+Tub). Data are presented as the means ± SD from three independent biological replicates. Student's *t* test was used for significant differences. * $P < 0.05$; ** $P < 0.01$.

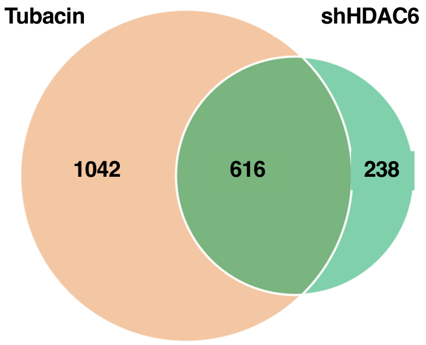

Figure S11. The effect of tubacin and HDAC6 knockdown on the radiation-induced upregulated genes. The Venn diagram shows the overlap between the gene set of RT-induced genes suppressed by tubacin and the gene set of RT-induced genes suppressed by the knockdown of HDAC6.

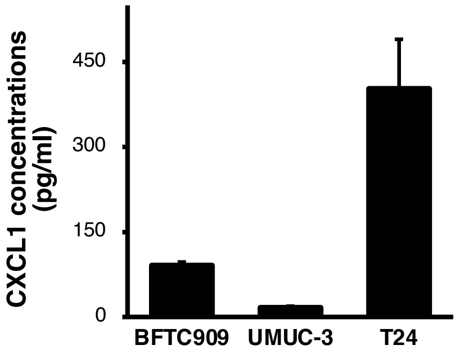

Figure S12. CXCL1 protein expression in human bladder cancer cell lines. CXCL1 protein levels in conditioned medium from BFTC909, UMUC-3 and T24 cells were measured by ELISA. Data are presented as the means ± SD from three independent biological replicates.

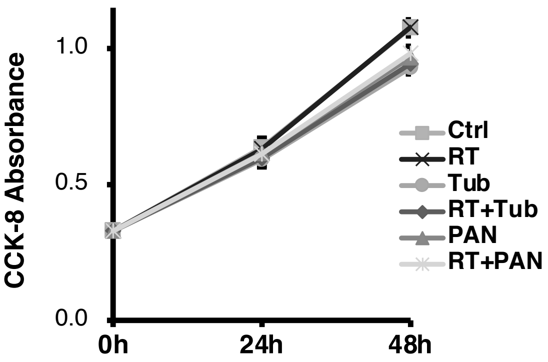

Figure S13. Quantification of viable cells under radiation, tubacin, panobinostat and combination treatment. Growth curves of T24 cells with or without 5 μM **tubacin** (Tub) treatment followed by 5 Gy irradiation (RT) were determined by the CCK-8 assay at 24 and 48 hours. Panobinostat (PAN, 10 nM) was used for comparison. Data are presented as the means ± SD from three independent biological replicates.

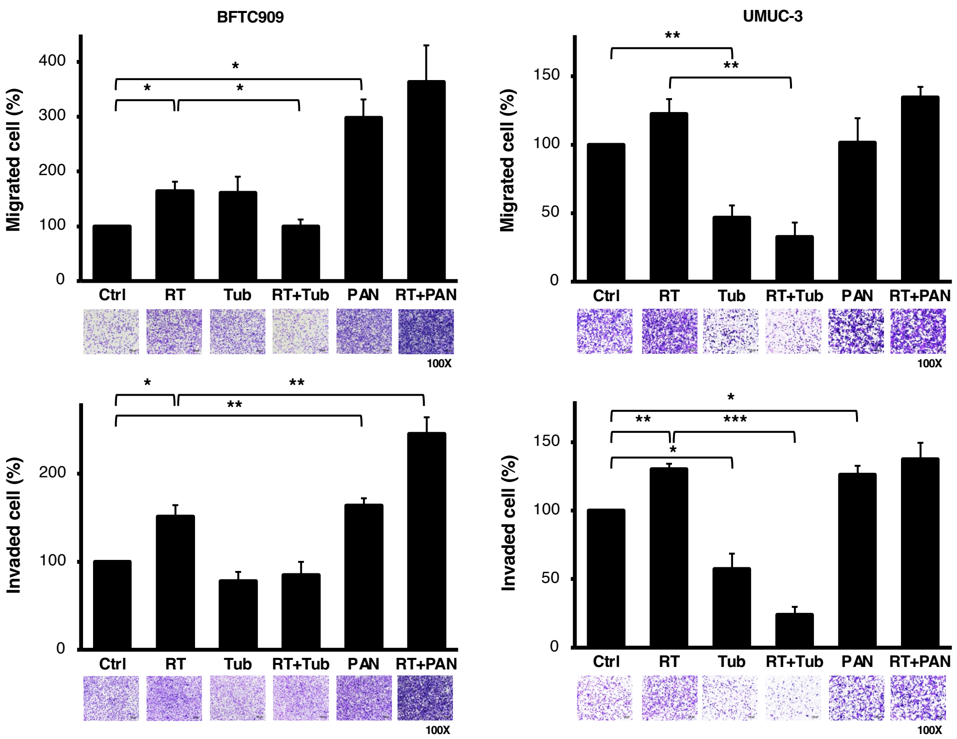

Figure S14. Tubacin combined with RT repressed the RT-induced migration/invasion of BFTC909 and UMUC-3 cells. Migration and invasion assays of BFTC909 and UMUC-3 cells treated with or without tubacin (Tub) followed by 5 Gy irradiation (RT). Migrated and invaded cells on the lower surface of the Transwell membrane were stained and counted at 16 and 20 hours, respectively. Representative images are shown below individually. Panobinostat (PAN) was used for comparison. Data are presented as the means ± SD from three independent biological replicates. Student's t test was used to determine significant differences. * $P < 0.05$; ** $P < 0.01$; *** $P < 0.001$.

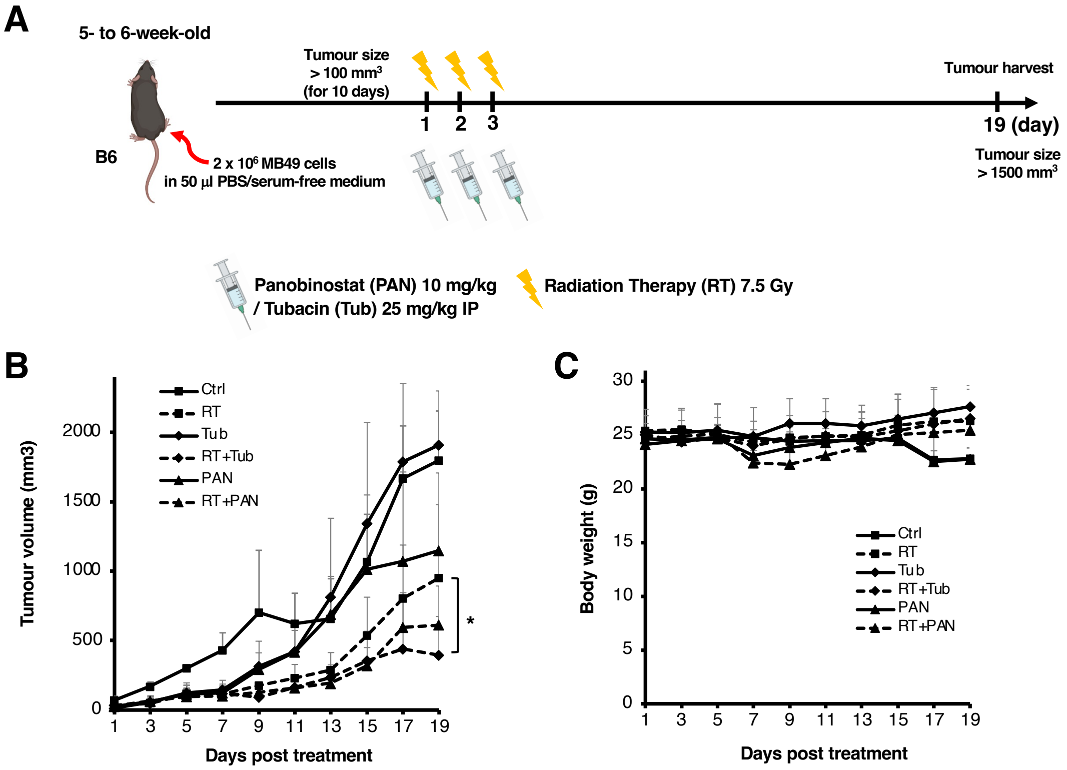

Figure S15. The HDAC6i tubacin exerts a radiosensitizing effect on tumor growth in vivo. **a** Timeline of the analysis of the ectopic tumor model. MB49 cells were injected subcutaneously into the right hind limb of thirty C57BL/6 mice. The experimental group was unexposed or exposed to 7.5 Gy of RT, and tubacin or panobinostat was administered by intraperitoneal injection for three days. Mice were sacrificed on day 19. **b** Tumor volumes were measured for each desired treatment group on the indicated days (n=5 for each group). **c** The body weight of each mouse group was recorded after the desired treatments on the indicated days (n=5 for each group). Data are presented as the means ± SD for each independent biological replicate. Student's t test was used to determine significant differences. * $P < 0.05$.

Table S1. List of antibodies, related to experimental procedures

Used for	Official Name	Cat. #	Distributor
WB	Acetyl-α-Tubulin (Lys40)	#5335	Cell Signaling
	Acetyl-Histone H3 (Lys9)	#9649	Cell Signaling
	Akt	#9272	Cell Signaling
	E-Cadherin	#14472	Cell Signaling
	GAPDH	#2118	Cell Signaling
	HDAC1	#5356	Cell Signaling
	HDAC2	#5113	Cell Signaling
	HDAC3	#3949	Cell Signaling
	HDAC4	#7628	Cell Signaling
	HDAC5	#20458	Cell Signaling
	HDAC6	#7558	Cell Signaling
	N-Cadherin	#13116	Cell Signaling
	p21 Waf1/Cip1	#2946	Cell Signaling
	Phospho-Akt (Ser473)	#9271	Cell Signaling
	Phospho-PTEN (Ser380/Thr382/383)	#9549	Cell Signaling
	PTEN	#9188	Cell Signaling
	Rad51	#8875	Cell Signaling
	Snail	#3879	Cell Signaling
	Vimentin	#5741	Cell Signaling
IF	Phospho-Histone H2A.X (Ser139)	#9718	Cell Signaling
IHC	GROα	sc-514065	Santa Cruz
Neutralized	Human/Primate CXCL1/GROα/KC/CINC-1	MAB275	R&D Systems

Table S2. List of primers

Gene Symbol	Forward 5'-3'	Reverse 5'-3'
CD58	TGT GCT TGA GTC TCT TCC ATC	ATT GCT CCA TAG GAC AAT CCC
CD274	CCT CAA GTG TCT GTG CAG TAT C	TAT CAC AAC AGG GTG GTT ACA G
CDKN1A	CGC TCT ACA TCT TCT GCC TTA G	CGG GAT GAG GAG GCT TTA AAT A
CXCL1	CAC AGT GTG TGG TCA ACA TTT C	AGT AAA GGT AGC CCT TGT TTC C
CXCL16	CAT CTT CAT CCT CAC CGC AG	CCT ATA ATC CTC GCA CCT TCA G
EDN1	TGG TAA CTG CTT TGG TCT CTT C	TTC TCC CTG AAA TGT GCC AG
GAPDH	CCA CTC CTC CAC CTT TGA C	ACC CTG TTG CTG TAG CCA
HDAC6	GTC TAC TGT GGT CGT TAC ATC	GGC CTG ACA GTA GTA ACA C
ITGA6	CAC AGG TTC TCA AGG GTA TAT CAC	AAC AGC AAC ATC AGG GTA GG
KIT	ACA TAG ACC CAA CAC AAC TTC C	GCT TCC CGT TCT GTC AAA TG
KRT7	GTG GTG GAG GAC TTC AAG AAT AA	CAT TTG GCC ATC TCC TCA TAC T
SDC1	CCC CAT CTT GCT TCC CTA ATC	CAG ATA GTC CAT ACC CTG TTG C
SDC2	GCC TGT CGA TGA GAC TGT TTA T	GGC AGC CTT CCA ATA TCT CTA C
SERPINE1	GCC CTT GAG TGC TTG TTA GA	GGT GAC ACA GAC CAC AAA GA
TFDP1	GCT CTG CCG AAG ACC TTA AA	GAG AAC CTT GTG CCG TTA GA
TGFB2	CTA CCC TAA GCG AGC AAT TCC	TCC AAA CGC CAA CCC AG
THBS1	GAG ACG GTA TCC TCA ATG AAC G	TCT CCC TTG CCA TCT TTG TC
TNFRSF1A	GAA GAA CCA GTA CCG GCA TTA	TCC TCA GTG CCC TTA ACA TTC
VEGFC	GAC CCC ACA AAG AAC TAG ACA G	CAT TCA CAG GCA CAT TTT CCA G
